# A randomized double-blind, placebo-controlled, cross-over trial (Vestparoxy) of the treatment of vestibular paroxysmia with oxcarbazepine

**DOI:** 10.1007/s00415-017-8682-x

**Published:** 2017-11-27

**Authors:** Otmar Bayer, Tatiana Brémová, Michael Strupp, Katharina Hüfner

**Affiliations:** 1 German Center for Vertigo and Balance Disorders, Campus Großhadern, Munich University Hospital, Marchioninistrasse 15, 81377 Munich, Germany; 2 Department of Neurology, Munich University Hospital, Munich, Germany; 3 Department of Psychiatry, Psychotherapy and Psychosomatics, University Hospital of Psychiatry II, Medical University Innsbruck, Innsbruck, Austria

**Keywords:** Vertigo, Randomized controlled trial, Cross-over studies, Class II Level of evidence, Vestibular paroxysmia, Neuro-vascular cross compression, Oxcarbazepine

## Abstract

**Objective:**

Vestibular paroxysmia (VP) is characterized by short, often oligosymptomatic attacks of vertigo which occur spontaneously or are sometimes provoked by turning the head. Despite the description of the disease almost 40 years ago (first termed “disabling positional vertigo”), no controlled treatment trial has been published to date. The Vestparoxy trial was designed as a randomized, placebo-controlled, double-blind cross-over trial to examine the therapeutic effect of oxcarbazepine (OXA) in patients with definite or probable VP.

**Methods:**

Patients were recruited from August 2005 to December 2011 in the outpatient Dizziness Unit of the Department of Neurology of the Munich University Hospital, and randomized to receive OXA (first week: 300 mg once per day, second week: 300 mg b.i.d., third week: 300 mg t.i.d. until the end of the third month), followed by placebo or vice versa with a 1-month wash-out period in between. The primary endpoint was the number of days with one or more attacks. Secondary endpoints were the number of attacks during the observed days, and the median (for each day) duration of attacks. All these endpoints were assessed using standardized diaries collected at the end of each treatment phase.

**Results:**

Forty-three patients were randomized, 18 patients provided usable data (2525 patient days) for at least one treatment phase and were included in the main (intention-to-treat) analysis. The most common reasons for discontinuation documented were adverse events. The risk of experiencing a day with at least one attack was 0.41 under OXA, and 0.62 under placebo treatment, yielding a relative risk of 0.67 (95% CI 0.47–0.95, *p* = 0.025). The number of attacks during the observed days ratio was 0.53 (95% CI 0.42–0.68, *p* < 0.001) under OXA compared to placebo. Median attack duration was 4 s (Q25: 2 s, Q75: 120 s) under OXA, and 3 s (Q25: 2 s, Q75: 60 s) under placebo treatment. When days with no attacks, i.e., duration = 0, were included in the analysis, these figures changed to 0 (Q25: 0, Q75: 3 s), and 2 (Q25: 0, Q75: 6 s). No serious adverse events or new safety findings were identified during the trial.

**Conclusions:**

The Vestparoxy trial showed a significant reduction of VP attacks under OXA compared to placebo treatment, confirming the known and revealing no new side effects.

## Introduction

Vestibular paroxysmia (VP) is characterized by recurrent, brief, mono- or oligosymptomatic attacks of vertigo which occur spontaneously or are rarely triggered by a provoking factor, most commonly a head or body turn [1, 2]. The condition was first described in 1975 along the lines of trigeminal neuralgia and hemifacial spasm, and was termed “disabling positional vertigo” [3, 4]. The initial nomenclature reflects the fact that affected patients are often severely disabled and cannot work or participate in daily activities. Since then some case reports and case series on the condition have been published, e.g., [5, 6], but no randomized controlled trials have been carried out.

The clinical diagnostic criteria of VP were first provided by Brandt and Dieterich [6] and then refined by Hüfner [1]. Although the disease is recognized as a diagnosis in most major dizziness centers (see [1, 7] for details) the absence of a gold standard diagnostic test has evidently hindered treatment studies on the topic. Most available clinical studies focus on the ancillary investigations, namely imaging data [1, 8]. It is now widely accepted that neuro-vascular cross compression (NVCC) of the eighth nerve leading to demyelination with ephaptic depolarization is the most probable underlying pathomechanism [9], although the finding of asymptomatic NVCC has been reported [10]. NVCC of the eighth nerve could be detected in all VP patients, resulting in a sensitivity of 100% and a specificity of 65% for the diagnosis of VP by MRI [8]. The vessels are mainly arteries, most often the anterior inferior cerebellar artery (AICA) [1, 8]. No structural lesions of the vestibular nerve were identified in 7 T MRI despite the presence of NVCC [11]. Audiovestibular testing has shown heterogeneous results ranging from normal results to unilateral deficits and coinstantaneous signs of reduced and increased function within the same nerve [1, 8].

In terms of treatment, in the initial studies, surgical decompression was recommended [12, 13]. Nowadays, the treatment of choice for VP, as with the other cranial nerve NVCC syndromes, is the use of carbamazepine (CBZ) or OXA, also based on two retrospective studies [1, 14]. Both showed a good effect of CBZ and oxcarbazepine (OXA) (for review see [15]).

Despite the description of the condition more than 40 years ago, no controlled treatment trial has been published to date. Therefore, we conducted a prospective randomized, double-blind, placebo-controlled cross-over study of OXA in VP. We chose OXA over CBZ because it is supposed to have a better neurotoxicity profile, leading to an advantageous risk–benefit profile superior to CBZ [16]. We aimed to assess whether OXA therapy is superior to placebo regarding attack frequency and duration. We also evaluated how OXA was tolerated in this specific patient population by monitoring adverse events throughout the study.

## Subjects and methods

### Protocol

The Vestparoxy trial was designed as a monocenter, placebo-controlled, cross-over trial, with patients randomized in a 1:1 ratio to receive placebo followed by OXA or vice versa.The trial was registered under EudraCT no. 2004-003395-10; the protocol was approved by the ethics committee of the University of Munich (project no. 285/04) as well as the competent national authority. The trial was performed in accordance with the Declaration of Helsinki and its subsequent amendments, as well as with the ICH Guideline for Good Clinical Practice.

### Subjects

Patients between 18 and 80 years of age with *definite* or *probable* VP were eligible, as defined by the following modified diagnostic criteria [6]: short spells of vertigo lasting from seconds to a few minutes; disturbance of gait or postural instability during the attacks; attacks occur at rest or can be provoked by hyperventilation, certain head positions or changes in head position; no central ocular motor disorder; treatment response to antiepileptic drugs (not applicable at presentation); additional criteria: reduced hearing or unilateral tinnitus during the attacks or permanently; increase of detectable vestibular/cochlear deficit during the course of the disease. *Definite* VP was identified when four diagnostic criteria or three plus one additional criterion were fulfilled; *probable* VP was identified when three diagnostic criteria or two plus one additional criterion were fulfilled, and *possible* VP was identified in patients who fulfilled fewer criteria.

Exclusion criteria included additional vestibular or ocular motor disorders such as benign paroxysmal positional vertigo or vestibular migraine at the time of initial presentation, and other neurologic disorders affecting the CNS. Pregnant or nursing women were not included.

Subjects were recruited from the outpatient Dizziness Unit of the Neurological Clinic of Munich University Hospital. Eligible subjects received written and oral information about the study, including possible side effects of OXA. Written informed consent was obtained from all subjects prior to inclusion.

The study was open for recruitment from August 2005 until December 2011. Last patient last visit (LPLV) was on November 10th 2011. The study was stopped because of the slow recruitment and the high number of drop-outs, since the minimum number of subjects stated in the protocol (at least 40 patients) was reached.

Participants were randomized to receive OXA in the first, and matching placebo in the second treatment period or vice versa. Each treatment period was 3 months long with a 1-month wash-out period in between. Study medication for the first period was dispensed at inclusion; for the second period, it was dispensed at the 2nd visit, scheduled during the washout phase. The 3rd and last visit was scheduled after the 2nd treatment period. Study medication was administered in an ascending therapeutic scheme (first week: one 300 mg tablet per day, second week: 300 mg b.i.d., third and subsequent weeks: 300 mg t.i.d.). Patients received written and oral instructions on how to take the medication and were asked to record the number of pills taken in the vertigo diary.

#### Patients’ self-report of attacks

Participants were instructed to document vertigo attacks, duration, intensity (1–10, one being the lowest), and accompanying symptoms in structured diary forms collected at each visit. They were asked to also enter days without symptoms by just filling in the date.

#### Monitoring of side effects

Side effects were assessed by handing out standardized forms to the patients, listing the known side effects of OXA (from the Tegretal^®^ SmPC, September 2004) ordered by organ system, along with instructions and contact information. As the electrolyte blood levels (especially sodium), blood count and liver function might change under therapy with sodium channel blockers, they were examined weekly for the first month and monthly for the rest of each treatment period by the patients’ general practitioner. At the follow-up visits, the forms were collected, and subjects were also asked if they had had any (including unexpected) side effects or no side effects at all. In the event of severe side effects or any kind of rash, patients were instructed to contact the investigators immediately.

In the protocol, attack frequency, duration, intensity, triggers and concomitant symptoms, as well as adverse reactions were listed as main outcomes. With regard to data quality of the patient-reported outcomes (see Data management), and to multiple testing, it was decided before unblinding the treatment allocation to reduce outcomes and to define the endpoints as follows: primary endpoint is the number of days with one or more attacks among the observed days (ATD). Secondary endpoints are: number of attacks during the observed days (NAT); median (for each day) duration of attacks. Adverse events are reported descriptively. The endpoints are compared for OXA versus placebo treatment period (including the time under ascending dose).

The sample size was set to at least 40 patients according to the protocol, the significance level was set to alpha = 5%.

#### Randomisation, concealment, and blinding

A simple randomisation with a computer-generated list of random numbers, which was generated in the pharmacy, was used to allocate the participants.

OXA (Tegretal^®^, Novartis Pharma GmbH, Nuremberg) 300 mg tablets were over-encapsulated with lactose monohydrate as filling material at the pharmacy of the Munich University Hospital. Identical capsules with the same filling material were used as placebo. OXA- and placebo-capsules were packed in bottles labeled with the random numbers and the order in which they should be taken (e.g., 14 A and 14 B together making up a kit for one participant), according to the randomisation list.

Participants were enrolled and examined by physicians from the neurologic outpatient clinic, mostly by one of the authors (KH). The investigational medical product was kept in numbered kits, which were assigned to the participants in ascending order.

Investigators and patients were blinded to the treatment allocation sequence.

### Data management

For analysis, a relational HSQL-Database (version 1.8, front end LibreOffice version 4) was set up and data from (paper) records, vertigo diaries, and adverse event (AE) collection forms were entered. Before data entry, written rules were set up and trained, to ensure consistent handling. This was particularly important for the diaries, which were not always completed by the participants as expected. To summarize, the database form allowed entry of quantitative (preferred where available) or qualitative measures of attacks per day. Duration was entered as the median duration of attacks on each day (in case different durations were recorded by the patient). Since patients did not always document attack-free days, documentation periods (several per patient possible, independent of treatment period) were classified as “attack-free days documented” or “attack-free days not documented”, and entered in the database. This was done to obtain information on data quality to support the blinded decision about confirmatory and exploratory endpoints, and to facilitate ancillary analyses. After data entry, verification against the source data was performed, and queries were generated by a second person, which were recorded in the database along with the answers.

The standardized side effect forms contained a field to record the onset date and duration (rarely documented) of the events, and date fields to specify the time period covered (start and end date). The latter allowed the event to be assigned to the treatment phases according to the following procedure: Forms with an end date not earlier than the start of the first treatment phase and before the start of the second treatment period were assigned to the first treatment period. Forms with an end date not earlier than the start of the second treatment period and at least 14 days after the end of the first treatment period were assigned to the second treatment period. Forms with an end date not earlier than the start of the second, and not more than 14 days after the end of the first treatment period were assigned to the first and/or second treatment period, i.e., OXA and/or placebo (see Table 2).

**Table 1:** Due to the high drop-out rate, this table gives population descriptives for the periods analyzed

OXA periods	Placebo periods
Age 20–73 years, median 52 years, mean 50 years	Age 20–73 years, median 56 years, mean 52 years
Gender: 6 females, 10 males	Gender: 5 females, 9 males

**Table 2:** Frequency of adverse events categorized by organ system

System	Symptom	Frequency		
		OXA	Placebo	OXA and/or placebo
General	Fatigue, feeling weak	5	3	2
	Swelling of eye lids or other parts of the body	2	1	–
Immune system	Symptoms of hypersensitivity involving more than one symptom or organ	2	–	–
Skin	Rash	1	–	–
CNS	Vertigo, dizziness	6	4	1
	Headache	6	4	3
	Nausea	5	1	2
	Disturbance of concentration	1	2	–
	Listlessness	1	–	1
	Restlessness	1	2	–
	Emotional instability	2	1	–
	Tremor	2	–	–
	Coordination impaired	1	–	–
	Nystagmus	–	1	–
Visual disturbances		3	–	1
Cardiac disorders	Rate and rhythm disturbances	–	–	1
Gastrointestinal disorders	Abdominal pain	2	1	1
	Obstipation	1	1	–
	Diarrhea	3	1	–
Hepatobiliary disorders		1	1	–
Metabolism disorders	Including sodium imbalance	1	1	1

Frequency gives the number of patients in whom the event occurred under the respective treatment. Multiple occurrences of the same symptom within one patient and treatment phase were counted as 1

### Statistical analyses

Statistical analysis and figure design was performed using R (version 3.3.2, www.r-project.org), except for modelling results which were obtained using PROC GENMOD in SAS 9.4 University Edition, SAS Institute, Cary USA. To facilitate reproducible analyses StatWeave (version 0.91_03, www.stat.uiowa.edu/~rlenth/StatWeave), a literate programming tool was used.

The classical approach for cross-over trials is to take the difference in the outcome parameter between active OXA treatment and placebo to test efficacy. However, this requires data for both treatment periods. Due to the high number of probably informative missings (resulting from drop-outs, periods of insufficiently filled in diaries), excluding such patients might introduce bias. Therefore, we decided before unblinding treatment allocation, to employ analyses with treatment (OXA vs. placebo) as grouping variable capable of including data from one or two treatment periods. Furthermore, this approach avoids reducing diary data by averaging to one number per patient and treatment period, which would raise weighting issues.

For the primary endpoint ATD and for the secondary endpoint NAT a Poisson regression model was used. The models were estimated applying the GEE approach (generalized estimating equations) [17]. GEE use quasi-likelihood to fit the model, account for clustered data (data within patients are not independent), and estimate a scaling parameter adjusting for overdispersion which can occur in Poisson regression. The results of median attack duration are shown descriptively.

## Results

**Fig. 1:**
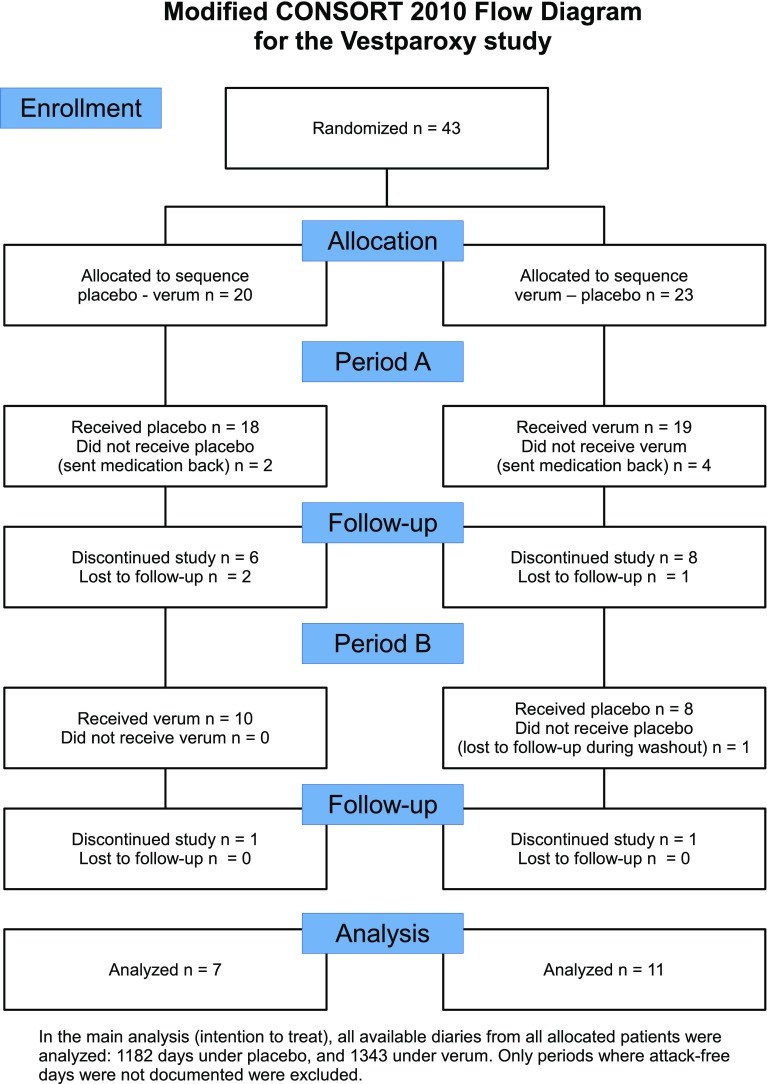
Participant flow

Patient flow is illustrated in Fig. 1. Forty-three patients were randomised, 20 to the placebo-verum, and 23 to the verum-placebo sequence. There were a high number of patients who discontinued the study during or after the first treatment period (see Period A in the figure), while patients mostly remained in the study, once they reached the second period. The most frequent reasons for discontinuation were AEs (*n* = 10), relief of symptoms (*n* = 2), and no effect (*n* = 2). Twenty-five patients delivered any diary data on a total of 3612 days. In the main analyses only diary periods classified as “attack-free days documented” (see Data management) were used, yielding 1343 days under OXA, and 1182 under placebo from 18 patients; 12 of them contributed data to both OXA and placebo period. The remainder was outside a treatment period (725 days, usually during wash-out) or not documented as just described.

Mean age was 61 (min. 20, max. 73) years; 6 of the 18 patients were female (see Table 1 for further details).

The risk of experiencing a day with at least one attack (ATD) was 0.41 under OXA, and 0.62 under placebo treatment, yielding a relative risk of 0.67 (95% CI 0.47–0.95, *p* = 0.025).

**Fig. 2:**
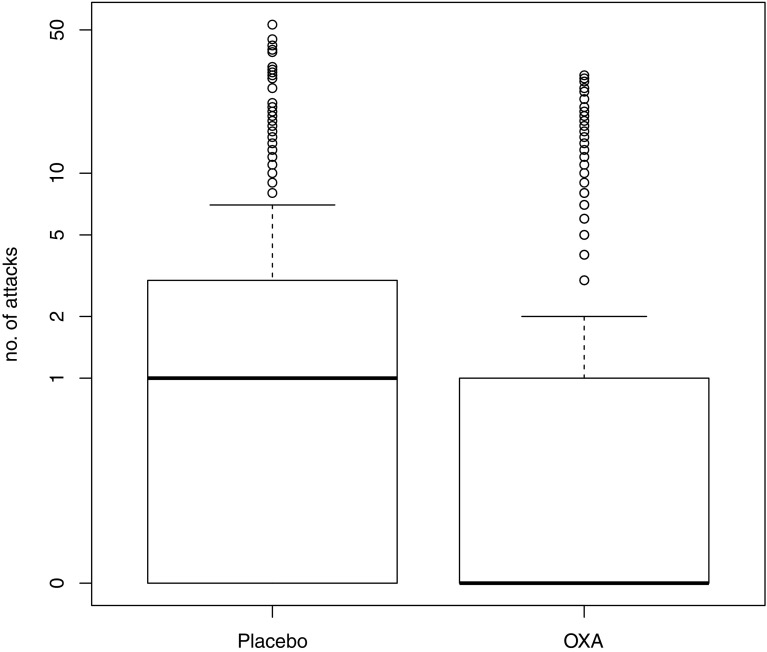
Boxplot of the number of attacks under placebo and OXA, *y*-axis scaled logarithmically, the bold black line and the box give the median and interquartile range of the distribution

The average number of attacks per day (NAT) was 3.15 under OXA, and 5.91 under placebo treatment, yielding a relative rate of 0.53 (95% CI 0.42–0.68, *p* < 0.001)(Fig. 2). While qualitative data (e.g. “many attacks”, “no attacks”) suffice to compute ATD, modelling the mean NAT requires quantitative data (e.g. “2 attacks”), reducing the main analysis set described above to 1098 + 902 days (OXA + placebo) from 18 patients.

Median attack duration (from 18 patients) was 4 s (Q25: 2 s, Q75: 120 s, *n* = 493 days) under OXA, and 3 s (Q25: 2 s, Q75: 60 s, n = 628 days) under placebo treatment. When days with no attacks, i.e., duration = 0, were included in the analysis, these figures changed to 0 (Q25: 0, Q75: 3) s, and 2 (Q25: 0, Q75: 6).

### Ancillary analyses

To check for the robustness of our result, we repeated the analysis of the primary endpoint ATD using logistic regression modelling the odds of ATD/attack-free days. The resulting odds ratio of 0.43 (95 CI 0.20–0.92) confirmed the reduction of ATD by OXA compared to placebo. We decided to keep the pre-specified analysis with Poisson regression, which fits the structure of the data better, and returned a better fit statistic (QIC -323 for Poisson, 3581 for logit).

Regarding attack duration, we hypothesized that there might be an effect of OXA on longer attacks (for pathophysiological reasons or detectability: if attacks last only for a few seconds, a further shortening might be unnoticed by the patient). This would not result in a shift of the whole duration distribution but only of its upper parts, leading to a false negative study result. We therefore ran quantile regression as an exploratory analysis, modelling the effect of the drug on Q75 of the duration distribution [18, 19]. However, OXA was associated with longer attack duration in this analysis.

### Harms

Adverse events were collected from all available forms, regardless of whether the patients contributed evaluable days for the efficacy analysis set. For some patients, the assignment of the adverse events to one of the two treatment phases was not possible (e.g., because the patient returned only one form covering the whole study period). The assignment procedure is described in Data management; the results are listed in Table 2. More adverse events occurred under OXA than under placebo treatment. Of the symptoms not listed on the adverse event form, tinnitus was the most frequently reported [as free text, OXA: (2), placebo: (3), OXA and/or placebo: (1)]. No pregnancies or serious adverse events occurred in the course of the trial.

## Discussion

In a randomized controlled clinical trial, OXA treatment reduced the number of days with at least one vertigo attack by one-third, and the average number of attacks almost to half the numbers under placebo. This is consistent with open-label studies and clinical experience with OXA and other sodium channel blockers in VP [1, 14]. This is also consistent with the benefit to patients with other neurovascular compression syndromes, i.e., trigeminal neuralgia and hemifacial spasm, from CBZ or OXA.

Median attack duration on days with attacks was increased under OXA with 4 s compared to 3 s under placebo. This might be accounted for by the inability of patients to distinguish VP attacks from (longer-lasting) dizziness arising as a side effect of OXA sometimes. When durations > 5 min were excluded, according to the current diagnostic criteria for probable VP [2], the difference diminished. When days without attacks, i.e., duration = 0, were included, the duration was smaller under OXA compared to placebo (even when durations > 5 min were not excluded), as reported in Results.

The most important limitation of the Vestparoxy trial is its high drop-out rate. Apart from patients refusing further participation before starting to take the study medication, AEs and relief of symptoms were the most frequent reasons for premature discontinuation. We compared the documented reasons under OXA and placebo treatment suspected of introducing bias to the study results: two patients aborted the study because of lack of efficacy (both under placebo), ten stopped because of AE (6 under OXA, 3 under placebo, 1 unclear). This points to side effects of OXA, but not to an overestimation of its treatment effect. When looking at the high number of drop-outs in the Vestparoxy trial, there is a substantial number because of AE, requiring to balance AEs against the reduction of attacks in the individual patient. However, there are also other or unknown reasons for discontinuation. It should be considered that a clinical trial requires a high level of motivation from patients, and leaving a clinical trial with its study visits, diaries, examinations, etc. is not the same as dropping a medication (potentially available for off-label use) outside a trial context.

In current trials, patient-reported outcomes regarding the subjective relief of symptoms or quality of life are often included. Unfortunately, no such variable is available for analysis in the Vestparoxy trial.

The quantitative assessment of vertigo remains a challenging issue. As there are no objective measurements available, researchers have to rely on patient reports. In the researchers’ experience with other studies in the field of vertigo, there is considerable variation in how patients fill in vertigo diaries. As described in Data management, considerable efforts were made to systematically capture the information provided by the patients in a study database. If we take the proportion of intervals classified as “attack-free days not documented” as a proxy for diary quality, this results in 9.7% (smaller is better) of all time documented under OXA & placebo for the whole study. For the main analysis, intervals classified as “attack-free days not documented” were removed, which reduced the statistical power, but is not expected to have differential effects on the two treatments.

In 2016, the diagnostic criteria for VP from the Classification Committee of the Bárány Society were published [2], defining definite VP and probable VP. For the diagnosis of VP a response to treatment with CBZ or OXA is now required, which makes this trial clinically even more important. They are similar to the criteria used in this clinical trial, and tend to be stricter. As in clinical routine patients sometimes do not fulfill all criteria for a disease, having evidence for the efficacy of a treatment tested in a population less strictly selected might be advantageous from a practitioners’ perspective. In general, the diagnosis applying the criteria is straightforward for clinicians experienced in vestibular disorders, and does not require extensive technical investigations.

Sodium channel blockers are regularly used to treat VP. As they are known to have side effects and require repeated laboratory testing, high-level evidence for their efficacy is desirable. The Vestparoxy study, a randomized controlled clinical trial, showed a substantial reduction of VP attacks under OXA treatment, confirming the known and revealing no new side effects.

## Abbreviations

AEs: Adverse events
AICA: Anterior inferior cerebellar artery
ATD: Number of days with one or more attacks among the observed days
CBZ: Carbamazepine
CI: Confidence interval
GEE: Generalized estimating equations
LPLV: Last patient last visit
NAT: Number of attacks during the observed days
NVCC: Neuro-vascular cross compression
OXA: Oxcarbazepine
VP: Vestibular paroxysmia
